# Sleep and breathing in children with cerebral palsy: it’s complicated….!*

**DOI:** 10.1016/j.jped.2025.101434

**Published:** 2025-08-11

**Authors:** David Gozal

**Affiliations:** Marshall University, Joan C. Edwards School of Medicine, Departments of Pediatrics and Biomedical Sciences and Office of the Dean, Huntington, USA

Children with cerebral palsy (CP) are at increased risk for sleep disorders, including obstructive sleep apnea (OSA), due to a multiplicity of factors related to their underlying condition. OSA, which is characterized by interruptions in sleep continuity along with increased upper airway resistance and collapsibility during sleep, can exacerbate existing health issues and impact the quality of life in children with CP in a phenotype-dependent fashion.^1^ Indeed, in a recent systematic review and meta-analysis, Nadeem and colleagues reported that in data originating from 42 studies using the Sleep Disturbance Scale for Children (SDSC), abnormal scores were present in 26 % (95 % CI: 17 %-37 %) of the children with CP. Furthermore, subscale abnormalities encompassed disorders of initiation and maintenance of sleep (28 %), sleep breathing disorders (17 %), excessive somnolence (12 %), sleep hyperhidrosis (10 %), and sleep-wake transition disorders (19 %).^2^ In another systematic review, the pooled prevalence of bruxism in children and adolescents with CP was 46% (95%CI: 0.38-0.55).^3^ Based on these findings and the clear interdependency of these two conditions, namely CP and sleep disorders, a more systematic exploration of their presence in clinical settings, aiming to improve the overall adverse impact of one condition on the other, would be clearly justified.

Among the factors potentially favoring the occurrence of sleep-disordered breathing in CP, the authors should enumerate several obvious ones: (a) Abnormal muscle tone affecting the muscles of the upper airway, leading to obstruction during sleep; (b) Craniofacial abnormalities that can contribute to airway narrowing; (c) Neurological and pulmonary issues involving respiratory control alterations, abnormal innervation and reflexes of the upper airway, swallow dysfunction leading to aspiration and upper airway inflammation and collapsibility, and hypertrophy of adenoid and tonsils; (d) Medications commonly used to manage CP symptoms can also increase the risk of sleep-disordered breathing; (e) Positioning difficulties that may favor upper airway restriction and difficulty controlling secretions leading to their accumulation within the oropharyngeal introitus; (f) Epilepsy and seizures, which if present may exacerbate OSA or be adversely affected by OSA; (g) The presence of chronic pain and spasticity may translate to disrupted sleep patterns that favor respiratory perturbations with reciprocal effects of sleep fragmentation on pain perceptual augmentation. Concurrent spasticity of the musculature, particularly in the muscles controlling upper airway patency and stability, can compromise normal breathing patterns both during wakefulness and sleep. However, implementation of interventions such as adenotonsillectomy, normally conducted as the first line of therapy for treatment of OSA in typically developing children, is not as likely to yield the anticipated beneficial outcomes in children with CP, thereby requiring an individually tailored clinical decision.^4^

Obviously, the presence of OSA in any child with CP is concerning by virtue of the adverse impact it can impose on the patient. Indeed, OSA has been associated with increased daytime sleepiness and behavioral changes such as irritability, hyperactivity, and difficulty concentrating, which can negatively affect learning and social interactions. Furthermore, underlying cardiovascular and respiratory disease risks can be worsened by the concurrent presence of OSA. Moreover, studies suggest that treating OSA may improve seizure control in some individuals with CP and epilepsy.^5^

Unfortunately, there are no CP-related specific tools that allow for robust screening of sleep disorders in this population.^6^ More importantly, studies that examine the potential changes in prevalence of OSA in children with CP based on the severity of their underlying disorder as concurrently assessed with previously validated functional scales such as the Gross Motor Function Classification System (GMFCS),^7^ the manual ability classification system and the communication function classification system^8^ are lacking. An additional severity classification system of particular relevance to the presence of upper airway dysfunction and risk of OSA would consist of assessments related to eating and swallowing^9^^,^^10^ in light of the deleterious impact that conditions such as drooling and sialorrhea may impose on sleep quality.^11^

In this issue of the Journal, a study encompassing 312 children assessed the prevalence of a high probability of OSA using a previously validated questionnaire^12^ and concurrently evaluated the corresponding GMFCS for each child.^13^ The overall prevalence of likely OSA was 9.0% in this cohort, with a particularly elevated frequency of putative OSA in those children with CP whose GMGCS scale scores corresponded to the most severe category, i.e., category V (14.7%). A valuable insight derived from this study, namely the confirmation of the GMFCS severity class V as the one bearing the highest risk of a variety of respiratory health issues in addition to OSA, is of importance, albeit not unexpected. Conversely, and in part noted by the investigators, the lack of objective sleep assessments and the implementation of a questionnaire tool that was not specifically developed for patients suffering from CP are important limitations. It is also unfortunate that other functional scales, particularly those related to drinking, eating, and swallowing, were not incorporated into the study. Notwithstanding such shortcomings, the study opens further the path towards future development of a multifactorial tool that enables accurate prediction of OSA and potentially other sleep problems in children with CP. It is clearly desirable that such an instrument, when available, can be longitudinally implemented in clinical settings to enable identification by clinicians of the optimal timing for referral to a sleep center for definitive diagnosis and treatment.

## Declaration of competing interest

The authors declare no conflicts of interest.
